# Multilevel socioecological correlates of physical activity, flourishing, and depression in children and adolescents: a structured evidence synthesis

**DOI:** 10.3389/fpubh.2026.1871867

**Published:** 2026-09-03

**Authors:** TaeEung Kim

**Affiliations:** Department of Golf and Leisure, Daegu Technical University, Daegu, Republic of Korea

**Keywords:** adolescents, children, depression, flourishing, narrative synthesis, physical activity, socioecological model, structured evidence synthesis

## Abstract

**Background:**

Childhood and adolescence are developmentally sensitive periods in which physical activity, positive mental health, and depressive symptomatology are associated with interacting socioecological conditions.

**Objective:**

This structured evidence synthesis summarized peer-reviewed evidence linking intrapersonal, interpersonal, organizational, community, and public-policy correlates with physical activity, flourishing, and depressive symptoms among youth aged approximately 6–18 years.

**Methods:**

Guided by PRISMA 2020 reporting items for transparent evidence synthesis, PubMed, Scopus, Web of Science, PsycINFO, and SPORTDiscus were searched for English-language peer-reviewed studies published from January 2000–April 2026. The review was not prospectively registered. Eligible records examined at least one socioecological correlate in relation to physical activity, flourishing, wellbeing, depression, or depressive symptoms. Data were extracted using a standardized form, and methodological quality was appraised using design-appropriate tools, including AMSTAR-2 for systematic reviews and meta-analyses.

**Results:**

Thirty-six evidence records were retained. Self-efficacy, enjoyment, perceived competence, parental logistic support, family cohesion, peer support, quality physical education, school sport opportunities, recreation-facility access, walkability, perceived safety, and national physical-activity policy emerged as recurrent correlates across socioecological levels. The evidence was most concentrated at intrapersonal and interpersonal levels and comparatively less developed for community and policy-level correlates. Evidence from prior meta-analyses suggested small-to-moderate beneficial associations of physical-activity interventions with depressive symptoms and broader mental-health outcomes.

**Conclusion:**

Youth physical activity, flourishing, and depression are associated with correlates distributed across multiple socioecological levels. Coordinated school, family, community, and policy strategies are needed to promote equitable physical-activity participation and positive mental health, while causal claims should be interpreted cautiously because much of the evidence remains observational.

## 1 Introduction

Childhood and adolescence are pivotal phases in the life course during which habits related to movement, emotional regulation, and social participation consolidate and track into adulthood (1). Regular physical activity (PA) confers well-documented benefits for cardiometabolic health, cognitive development, and mental wellbeing, yet global surveillance data indicate that approximately four in five adolescents fail to meet the World Health Organization (WHO) recommendation of at least 60 min of moderate-to-vigorous PA daily (2, 3, 26). In parallel, the global prevalence of adolescent depressive disorders has risen markedly, and mental disorders now account for roughly 13% of the overall burden of disease in people aged 10–19 years (4). Complementary developmental evidence also links physical activity in the early years and muscular fitness in children and adolescents with a broad range of health indicators (31, 32).

Beyond preventing ill-being, contemporary public-health discourse has embraced a positive-psychology framework emphasizing the cultivation of flourishing, a multidimensional construct integrating emotional, psychological, and social wellbeing (5, 6). Keyes (5) characterized mental health as a continuum ranging from languishing to flourishing and provided empirical evidence that flourishing is qualitatively distinct from the mere absence of mental illness. Huppert and So (6) extended this framework, identifying positive emotion, engagement, meaning, resilience, and positive relationships as the core pillars of flourishing. In pediatric populations, the Child Flourishing Index (CFI) operationalizes flourishing through three developmentally appropriate indicators: curiosity about learning, persistence in finishing tasks, and emotional self-regulation (7).

An ecological lens is useful for understanding how PA, flourishing, and depression are co-located within youth developmental contexts. Bronfenbrenner's bioecological theory (8) situates development within nested systems ranging from the proximal microsystem to the distal macrosystem, whereas McLeroy and colleagues' socioecological model (SEM) (9) operationalizes this nesting into five actionable strata (intrapersonal, interpersonal, organizational, community, and public policy), each of which may reinforce or constrain health behaviors. Sallis and colleagues (10) translated the SEM into an active-living framework that has since underpinned population-level PA surveillance and intervention design. Despite the ubiquity of this framework, reviews that consolidate evidence simultaneously across PA participation, flourishing, and depressive symptomatology in youth remain scarce.

Three observations motivate the present review. First, PA has been linked to significantly lower odds of languishing and substantially higher odds of flourishing in nationally representative samples of American adolescents (11, 12). Second, meta-analytic syntheses and reviews of reviews indicate small-to-moderate reductions in depressive symptoms following structured PA interventions, with effects intensified among adolescents older than 13 years and those with elevated baseline symptoms (13–15, 27). Third, ecological correlates at multiple levels, including parental logistic support, peer relationships, school physical-education (PE) quality, neighborhood-built environments, and national policy, are each independently related to youth PA (16–20, 29); however, their joint and interactive contributions to flourishing and depression are under-synthesized.

Against this backdrop, the present structured evidence synthesis pursues three aims. First, to map the SEM-level correlates of PA, flourishing, and depressive symptoms in children and adolescents. Second, to appraise methodological quality and identify sources of heterogeneity across the evidence base. Third, to articulate actionable implications for school-based, community-level, and policy-level strategies that integrate physical-activity promotion with mental-health cultivation in youth.

## 2 Methods

### 2.1 Protocol and reporting standards

This article is presented as a structured narrative evidence synthesis using systematic search and screening procedures. PRISMA 2020 (21) was used as a reporting framework to enhance transparency in record identification, screening, eligibility assessment, data extraction, and flow-diagram presentation; however, the work was not prospectively registered and should not be interpreted as a registered systematic review. Before screening, the author developed an internal protocol specifying the review question, eligibility criteria, information sources, data-extraction fields, quality-appraisal tools, and narrative-synthesis approach; this protocol was retained in an author-held audit file. No registration was completed in PROSPERO, OSF, INPLASY, or another public registry. Retrospective registration was not pursued because screening and synthesis had already commenced and retrospective registration would not provide the prospective transparency expected of a registered review. Methodological decisions made during the review process were documented in an author-held audit file. The review did not include unpublished material, submitted manuscripts, or personal communications.

### 2.2 Eligibility criteria

Studies were included when they sampled children or adolescents aged 6–18 years; measured at least one socioecological model-level correlate; reported quantitative or systematically synthesized associations with physical activity, flourishing, wellbeing, depression, or depressive symptoms; and were published as peer-reviewed English-language journal articles between January 2000 and April 2026. Editorials, dissertations, conference abstracts, non-English full texts, non-youth samples, and clinical-only samples without extractable youth data were excluded.

### 2.3 Information sources and search strategy

Five electronic databases were searched: PubMed, Scopus, Web of Science, PsycINFO, and SPORTDiscus. The Boolean search string combined concept blocks for population, physical activity or exercise, positive and negative mental-health outcomes, and socioecological correlates. Reference lists of eligible studies and prior systematic reviews were hand-searched. The full database-specific search strategies are provided in the Supplementary material. The search strategy is summarized in Table 1.

**Table 1:** Search strategy.

Component	Details
Databases	PubMed, Scopus, Web of Science, PsycINFO, SPORTDiscus
Date range	January 2000–April 2026
Language	English
Population block	child^*^ OR adolescent^*^ OR youth OR teen^*^ OR “school-aged”
Exposure block	socioecological OR socio-ecological OR Bronfenbrenner OR “social environment” OR “built environment” OR “parental support” OR “peer support” OR policy OR “physical education” OR neighborhood
Outcome block	“physical activity” OR exercise OR flourishing OR “wellbeing” OR depression OR “depressive symptoms”
Filters applied	Humans; peer-reviewed journal articles; age 6–18 years
Screening approach	Single-author screening, extraction, and quality appraisal using prespecified criteria; no inter-rater agreement statistic calculated

Hand searches of the reference lists of eligible studies contributed 47 additional records.

### 2.4 Study selection and data extraction

Records were de-duplicated in EndNote and uploaded to Rayyan for systematic screening. The author evaluated titles/abstracts and then assessed full texts against the eligibility criteria. For each included study, data were extracted into a standardized sheet capturing bibliographic identifiers, country, design, sample size, age range, SEM level(s) examined, exposure measures, outcome measures, and main findings.

### 2.5 Methodological quality appraisal

The Newcastle-Ottawa Scale (NOS) was used for cohort and case-control studies, whereas the AXIS critical-appraisal tool was applied to cross-sectional studies. For randomized and non-randomized trials, risk of bias was assessed with the Cochrane RoB 2 and ROBINS-I tools, respectively. Systematic reviews, meta-analyses, and reviews of reviews were appraised using AMSTAR-2 or AMSTAR-style criteria when the original design did not map directly onto AMSTAR-2. Qualitative and mixed-methods syntheses were appraised using MMAT-style criteria focused on sampling adequacy, analytic transparency, and integration of qualitative and quantitative evidence. Because this was a single-author review, inter-rater agreement was not calculated; quality appraisal was conducted using prespecified criteria, repeated consistency checks, and a study-level audit table before synthesis.

To reduce reviewer-dependent error, extracted fields and appraisal judgments were checked twice against the source records, and the full study-level quality summary was reported in the Supplementary material to maximize transparency for readers.

### 2.6 Data synthesis

**Figure 1:**
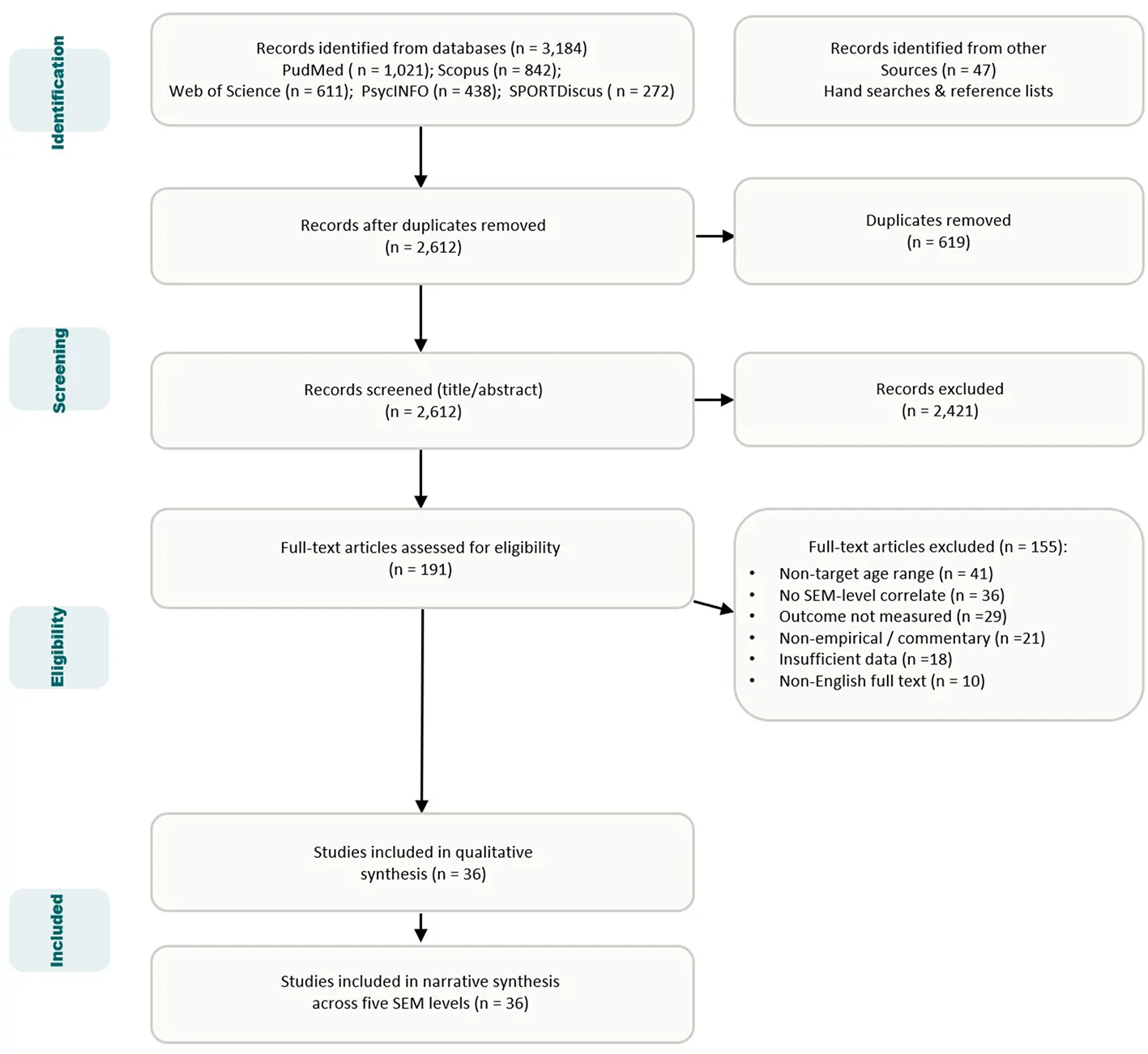
PRISMA 2020 flow diagram of records identified, screened, assessed for eligibility, and included in the evidence synthesis.

**Figure 2:**
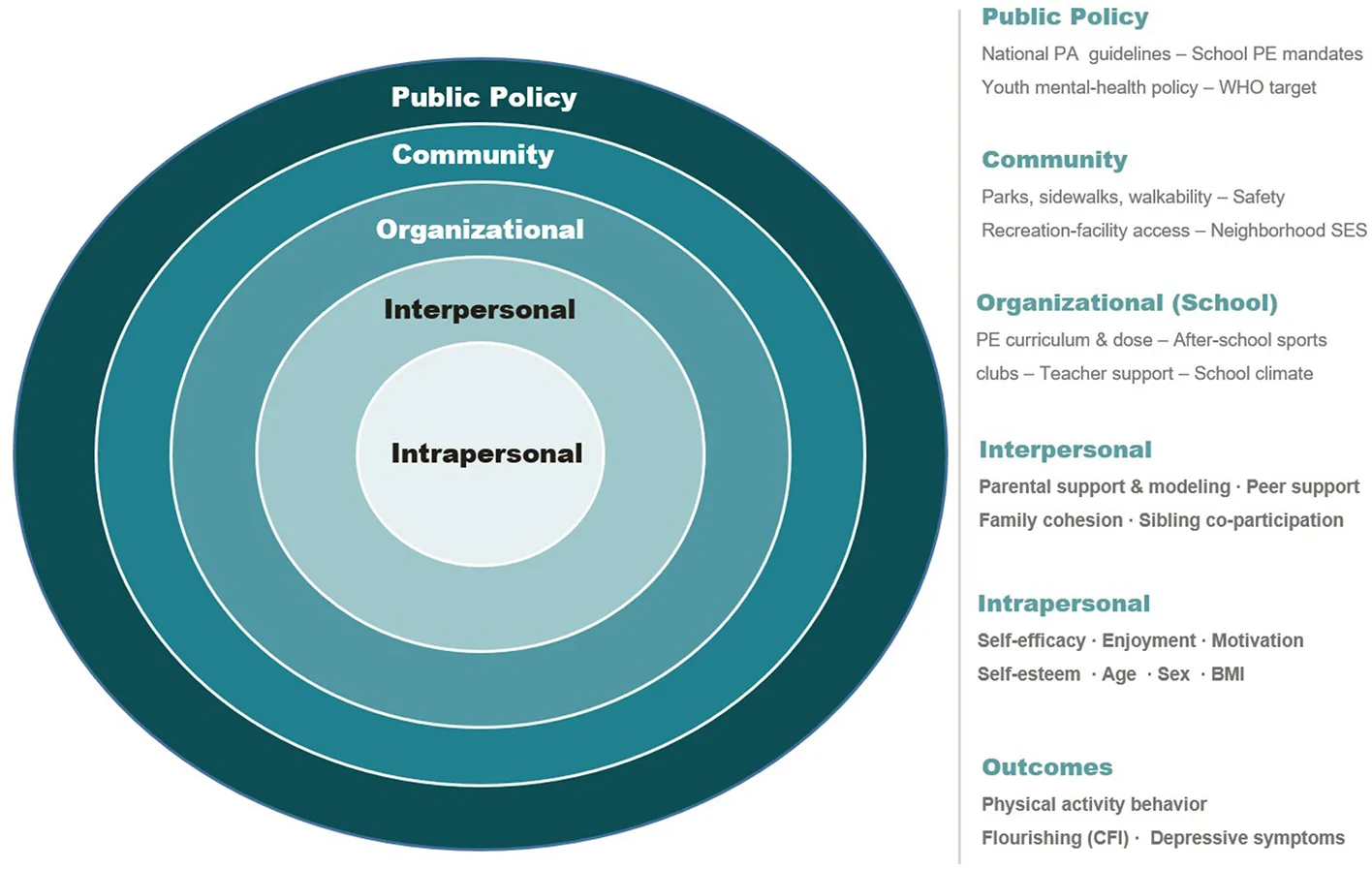
Five-level socioecological model adapted from McLeroy et al. (9) and Sallis et al. (10) and applied to children and adolescents' physical activity, flourishing, and depressive symptoms.

Given the expected heterogeneity in designs, measures, and populations, a narrative synthesis was prioritized over meta-analysis. Findings were grouped according to the five SEM levels. Pooled effect sizes from included meta-analytic reviews are reported descriptively alongside the primary evidence base. The PRISMA flow diagram summarizes the selection process (Figure 1), and the conceptual socioecological model guiding the synthesis is shown in Figure 2.

## 3 Results

### 3.1 Study selection and characteristics

The database search returned 3,184 records, with 47 additional records identified through hand searches (total 3,231). After removal of 619 duplicates, 2,612 records were screened at the title/abstract level, of which 191 proceeded to full-text assessment. Thirty-six evidence records were ultimately retained (Figure 1). The final evidence base included primary observational studies, longitudinal analyses, intervention trials, systematic reviews, meta-analyses, reviews of reviews, and narrative or umbrella syntheses relevant to youth PA, flourishing, wellbeing, depression, or depressive symptoms. Samples ranged from small school-based cohorts to nationally representative surveys, and records represented 19 countries across North America, Europe, East Asia, and Oceania. Table 2 summarizes the characteristics and principal findings of a representative subset of 15 records. Supplementary Table S1 provides study-level details for all 36 evidence records retained for qualitative synthesis, and Supplementary Table S1a presents the corresponding methodological-quality and risk-of-bias summary. A synthesis of findings across the five socioecological levels is presented in Table 3.

**Table 2:** Characteristics and principal findings of included studies (selected *n* = 15).

References	Country	Design/*N*	Age (y)	SEM level(s)	Primary outcome(s)	Key finding
Foster et al. (11)	USA	Cross–sectional/24,488	12–17	Intrapersonal	Flourishing	Adolescents meeting PA guidelines were 4.13 × more likely to flourish than inactive peers.
Bates and Hansen. (12)	USA	Cross–sectional/4,507	12–17	Intrapersonal	Flourishing/ academic engagement	Any PA, even below guideline levels, increased odds of flourishing and academic engagement in adolescents with obesity.
Bethell et al. (7)	USA	Cross–sectional/47,873	6–17	Interpersonal/ Community	Flourishing (CFI)	Family resilience and connection indices graded with flourishing across adversity levels.
Rodriguez–Ayllon et al. (13)	International	SR–MA/114 studies	2–18	Intrapersonal/ Interpersonal	Mental health (ill– and wellbeing)	PA effect on mental health ES = 0.17 (95% CI 0.11–0.24); significant for adolescents.
Recchia et al. (14)	International	SR–MA/21 RCTs	< 19	Intrapersonal/ Organizational	Depressive symptoms	PA interventions reduced depressive symptoms (g = −0.29; stronger for adolescents 13 years or older).
Wang et al. (15)	International	SR–MA/19 RCTs	12–18	Intrapersonal	Depressive symptoms	Exercise SMD = −0.64 (95% CI −0.89, −0.39); moderate intensity aerobic most effective.
Lubans et al. (16)	International	SR/25 articles	< 18	Intrapersonal/ Organizational	Cognitive & mental health	Improvements in physical self–perceptions accompanied self–esteem gains.
Hu et al. (17)	International	SR–MA/43 studies	5–18	Multi–level (SEM)	Non–organized PA	Overall ecological correlation r′= 0.32; macro/chronosystem r′= 0.50.
Ornelas et al. (18)	USA	Longitudinal/13,246	Grades 7–12	Interpersonal	MVPA	Family cohesion, communication, and engagement predicted MVPA; self–esteem mediated the path.
Tsuji et al. (19)	Japan	Cross–sectional/717	~9	Interpersonal	MVPA/light PA	Parental logistic support associated with higher MVPA; community resource use with walking.
Ding et al. (20)	International	SR/103 studies	5–18	Community	PA	Walkability, traffic safety, and recreation–facility access were most consistent correlates.
Christiana et al. (22)	USA	Cross–sectional/1,286	12–17	Intrapersonal × Community	MVPA	Supportive neighborhoods strengthened the psychosocial–MVPA relationship.
Kepper et al. (23)	International	SR/127 studies	5–18	Community	Active travel/PA/ well–being	Less traffic, better walkability, and shorter distances promoted active travel.
Gavand et al. (24)	USA	Cross–sectional/928	~14	Community	Accelerometer MVPA	Higher recreation-facility availability was associated with higher MVPA (~22 min/day difference between highest and lowest quintiles).
Van Sluijs et al. (3)	Global	Position paper/umbrella	10–19	Organizational/ Community/Policy	PA	Supportive schools, social environments, and urban design are priority policy levers.

CFI, Child Flourishing Index; ES, effect size; MVPA, moderate–to–vigorous physical activity; PA, physical activity; RCT, randomized controlled trial; SEM, socioecological model; SR–MA, systematic review and meta–analysis.

**Table 3:** Synthesis of evidence across the five socioecological levels.

SEM level	Representative correlates	Magnitude/direction of association	Illustrative sources
Intrapersonal	Self–efficacy, enjoyment, motor competence, motivation, self–esteem, age, sex	Moderate & positive for PA and flourishing; moderate & inverse for depressive symptoms	Foster et al. (11); Rodriguez–Ayllon et al. (13); Recchia et al. (14); Wang et al. (15); Lubans et al. (16)
Interpersonal	Parental logistic support, family cohesion, parent–child communication, peer support, family resilience	Consistent positive links with PA and flourishing; buffers depressive symptoms	Ornelas et al. (18); Tsuji et al. (19); Bethell et al. (7); Hu et al. (17)
Organizational	PE dose & quality, after–school sport, teacher support, school connectedness	Small–to–moderate positive effect on PA and mental health	Van Sluijs et al. (3); Lubans et al. (16); Recchia et al. (14)
Community	Walkability, recreation facility access, parks/green space, perceived safety, neighborhood SES	Moderate positive for PA; emerging positive for flourishing	Ding et al. (20); Kepper et al. (23); Gavand et al. (24)
Public Policy	National PA guidelines, GAPPA, school PE mandates, surveillance systems	Moderate & distal; often equal to or larger than microsystem effects	WHO (2); Van Sluijs et al. (3); Hu et al. (17)

GAPPA, Global Action Plan on Physical Activity; PA, physical activity; PE, physical education; SEM, socioecological model; SES, socioeconomic status.

### 3.2 Methodological quality and risk of bias assessment

Across the 36 included evidence records, methodological quality ranged from moderate to high, but certainty varied by study design and SEM level. Cross-sectional studies appraised with AXIS were generally rated moderate because they used large samples and clearly defined measures but were limited by single-time-point exposure and outcome assessment, self-reported PA, and residual confounding. Cohort and longitudinal studies assessed with the NOS were generally moderate-to-high quality, although attrition, unequal follow-up duration, and incomplete adjustment for screen time, socioeconomic status, and baseline mental health remained recurrent concerns. Randomized and quasi-experimental studies assessed with RoB 2 or ROBINS-I provided stronger intervention evidence but commonly showed some concerns related to allocation concealment, blinding, sample size, and short follow-up.

Systematic reviews and meta-analyses appraised with AMSTAR-2 were mostly moderate-to-high quality, particularly when they reported comprehensive search strategies, duplicate procedures, and quantitative heterogeneity assessment. However, some reviews were downgraded for limited reporting of excluded studies, incomplete certainty assessment, or high heterogeneity across PA and mental-health measures. Evidence was most robust for intrapersonal and interpersonal correlates, where large surveys, longitudinal analyses, and multiple meta-analyses converged. Organizational evidence was moderate but heterogeneous because school and after-school programs varied substantially in dose, implementation fidelity, and outcome measurement. Community-level evidence was moderate for built-environment correlates of PA but less consistent for flourishing and depression. Policy-level evidence was the least direct and was therefore interpreted as contextual and hypothesis-generating rather than causal. Table 4 summarizes the design-level appraisal, and Supplementary Table S1a provides the study-level assessment.

**Table 4:** Methodological quality and risk–of–bias appraisal across included evidence records (*N* = 36).

Design/evidence type (k)	Appraisal tool	Overall quality/risk level	Main methodological concerns and SEM–level implications
Cross–sectional and population surveys	AXIS	Moderate to high; risk of bias: some concerns	Large samples and clear measures strengthened intrapersonal/interpersonal evidence, but self–report PA, single–time–point design, and residual confounding limited causal inference.
Prospective cohort/longitudinal analyses	NOS	Moderate to high; risk of bias: low to some concerns	Temporality was stronger than in cross–sectional studies, but attrition, inconsistent follow–up, and incomplete adjustment for screen time, SES, and baseline mental health remained important limitations.
RCTs and quasi–experimental studies	RoB 2/ROBINS–I	Mixed; risk of bias: some concerns to moderate	Small samples, short follow–up, PE–teacher unblinding, and variable intervention fidelity limited the certainty of school and exercise–intervention evidence.
Systematic reviews/meta–analyses/reviews of reviews	AMSTAR−2 or AMSTAR–style criteria	Moderate to high; risk of bias: some concerns	Many reviews used comprehensive searches and heterogeneity assessment; downgrading reflected incomplete excluded–study lists, high heterogeneity, and limited certainty assessment.
Qualitative, mixed–methods, narrative or umbrella syntheses	MMAT–style or narrative relevance appraisal	Moderate to high relevance; risk of bias: interpretive	These records informed contextual interpretation of organizational, community, and policy–level correlates but were not treated as direct causal evidence.

AMSTAR-2, A MeaSurement Tool to Assess systematic Reviews 2; AXIS, Appraisal tool for Cross-Sectional Studies; MMAT, Mixed Methods Appraisal Tool; PE, physical education; RoB, risk of bias; SES, socioeconomic status.

### 3.3 Intrapersonal correlates

Psychological and behavioral determinants dominated the intrapersonal evidence base. Self-efficacy, enjoyment, perceived motor competence, autonomous motivation, and intention to be active were consistently associated with higher MVPA across cross-sectional and longitudinal studies (17, 22). Foster and colleagues (11) showed that adolescents who did not participate in any PA were 4.13 times less likely to flourish than peers meeting the 60-min daily guideline; even one to three PA days per week nearly doubled the odds of languishing. The dose-response appeared non-linear, with no significant additional benefit for daily versus four-to-six days per week of activity, suggesting a saturation threshold consistent with prior public-health analyses (3, 11). Consistent with this pattern, a systematic review and meta-analysis linked self-determined motivation with higher PA among children and adolescents (30).

Mental-health effects at the intrapersonal level were substantiated by two high-quality meta-analyses. Recchia and colleagues (14) synthesized 21 trials (*n* = 2,441) and reported that PA interventions significantly reduced depressive symptoms (Hedges' g = −0.29; 95% CI −0.47 to −0.10), with larger effects among adolescents 13 years or older and those with pre-existing clinical symptoms. Wang and colleagues (15) reported a larger pooled effect (SMD = −0.64) for moderate-intensity aerobic exercise, with three-to-four sessions per week over 6–8 weeks optimal. Mechanistic evidence from Lubans et al. (16) indicates that physical self-perceptions and self-esteem represent the most robust psychosocial mediators of the PA-mental health link in youth, while neurobiological and behavioral mechanisms remain underexplored.

### 3.4 Interpersonal correlates

The interpersonal stratum comprised family, peer, and sibling influences. In a 1-year longitudinal analysis of 13,246 Add Health adolescents, Ornelas and colleagues (18) demonstrated that family cohesion, parent-child communication, and parental engagement predicted achievement of five or more weekly MVPA bouts, with self-esteem mediating the pathway for both sexes and depressive symptoms mediating it among males. These findings align with Tsuji and colleagues‘ Japanese cohort (19) in which parental logistic support, particularly enrolment in sports clubs-predicted higher MVPA (β = 0.231, *p* < 0.001), whereas modeling and sedentary-behavior limiting were non-significant. Longitudinal evidence further indicates that parental encouragement and supervision predict children's time spent outdoors over time (28). Peer dynamics mattered in parallel. Hu and colleagues' meta-analysis (17) showed that peer social support was a stronger predictor of PA in girls than boys, and that best-friend PA modeling was significantly associated with children's participation.

Flourishing was also interpersonally patterned. Bethell and colleagues (7) documented a graded relationship between the Family Resilience and Connection Index (FRCI) and the CFI across household income and adverse-childhood-experience strata: children with the highest FRCI scores were 3.71 × more likely to flourish than those with the lowest. These results extend earlier findings that family warmth, autonomy-supportive parenting, and positive sibling relationships foster developmental strengths that buffer depressive risk (7, 18).

### 3.5 Organizational (School) correlates

Schools constituted the single most frequently studied organizational context. Quality physical-education (PE) provision, availability of after-school sport programs, and teacher support predicted both PA and broader mental-health outcomes. Evidence from adolescent PA syntheses and mechanism-focused reviews indicates that supportive school climates, quality PE, and structured sport opportunities may operate through psychosocial mechanisms such as self-efficacy, physical self-concept, and school connectedness. Hu et al. (17) confirmed positive associations between in-school sports, adult supervision, and MVPA, while Van Sluijs and colleagues (3) identified supportive schools as one of three pillars of effective adolescent PA ecosystems, along with social/digital environments and multi-utility urban design. School recess may also contribute to habitual activity, with sex differences in playground PA observed in school-aged children (33).

Intervention evidence at the organizational level has produced modest but meaningful effects. Prior reviews indicate that school and after-school PA programs are most promising when they combine structured activity with behavioral, social, and environmental supports, although effects vary by intensity, delivery context, and baseline mental-health status. Depression-specific intervention benefits appear greatest when programs include regular moderate-intensity aerobic activity, consistent with the Recchia (14) and Wang (15) meta-analyses.

### 3.6 Community correlates

Neighborhood and built-environment attributes emerged as the most consistent community-level correlates of youth PA. Ding and colleagues' (20) canonical review identified walkability, traffic speed/volume, access and proximity to recreation facilities, land-use mix, and residential density as the most supported environmental correlates across both children and adolescents. Subsequent evidence by Gavand and colleagues (24) showed that adolescents living in the top quintile of recreation-facility availability accumulated, on average, 22 min more daily MVPA than those in the lowest quintile. Kepper and colleagues (23) synthesized 127 studies and concluded that pedestrian infrastructure, shorter distances to destinations, and reduced traffic exposure supported active travel; the evidence for wellbeing and organized-sport outcomes was comparatively limited.

Community factors intersected with positive mental health. Foster and colleagues (11) observed that neighborhood amenities, including parks, sidewalks, and recreation centers, were significantly associated with flourishing, independent of PA participation. This is consistent with the broader public-health literature identifying equitable spatial distribution of green space and recreation infrastructure as an upstream determinant of psychosocial wellbeing (3, 20).

### 3.7 Public policy correlates

Policy-level evidence was sparser but conceptually important. The WHO Global Action Plan on Physical Activity 2018–2030 (GAPPA) (2) articulates 20 policy actions organized around four strategic objectives: creating active societies, environments, people, and systems, with a target of reducing physical inactivity by 15% by 2030. Van Sluijs and colleagues (3) emphasized that policy action should prioritize upstream drivers, including built environment, school policy, and social/digital environments, and underscored the inequitable distribution of supportive contexts, particularly in low- and middle-income countries. National surveillance analyses from the United States (11, 12) and multi-country comparisons (17) further suggest that macro-level factors, such as national PA policy, surveillance systems, and sport culture, are associated with youth PA patterns, although the available evidence does not permit strong causal inference at the policy level.

## 4 Discussion

The present review integrates evidence from 36 peer-reviewed evidence records to map socioecological correlates of PA, flourishing, and depressive symptomatology in youth. Because the evidence base is heterogeneous and includes many observational studies, the synthesis emphasizes patterns of association rather than causal effects. Four overarching insights emerge from the synthesis.

### 4.1 Cross-level convergence and the saturation threshold

First, correlates across all five SEM strata converge on daily PA, yet diverge in magnitude and developmental salience. Intrapersonal correlates such as self-efficacy and enjoyment were consistently associated with PA participation, but their predictive strength appears contingent on supportive interpersonal and community contexts. Christiana and colleagues (22) demonstrated that the association between friend support, norms, and attitudes with MVPA was strengthened in neighborhoods with greater PA-resource availability, exemplifying cross-level moderation. The saturation threshold identified in flourishing analyses (11), with no significant difference between daily and four-to-six-day PA participation, suggests that public-health messaging emphasizing any MVPA above a weekly baseline may be as consequential as promoting strict adherence to daily guidelines.

### 4.2 Flourishing as a distinct positive-mental-health outcome

Second, the synthesis reinforces the theoretical distinction between flourishing and the absence of depressive symptoms. Keyes (5) originally argued that flourishing is not simply the mirror image of mental illness; the present review supports this view. Bethell and colleagues (7) found that flourishing varied with family resilience and connection even after controlling for adverse childhood experiences, and Foster and colleagues (11) and Bates and Hansen (12) demonstrated incremental prediction of flourishing from PA beyond sociodemographic variables. Operationalizing flourishing through the CFI in pediatric populations expands the outcome space of PA research beyond pathology. However, the index is parent-reported and focused on three attributes, and future work should examine whether youth self-report instruments, such as the Mental Health Continuum-Short Form for adolescents, yield comparable patterns across SEM levels.

### 4.3 Mechanistic gaps and the primacy of psychosocial mediation

Third, psychosocial mediators, notably physical self-concept, self-esteem, school connectedness, and perceived competence, are the most empirically supported mechanisms linking PA to mental health. Lubans and colleagues (16) identified physical self-perceptions as the dominant pathway, whereas Ornelas and colleagues (18) established self-esteem as a mediator of the relationship between parental support and PA. School-based and ecological syntheses extend this interpretation by showing that supportive school environments and organized activity contexts provide proximal conditions through which PA-related psychosocial resources can develop (3, 16, 17). In contrast, neurobiological mechanisms (e.g., prefrontal cortex development, cerebral perfusion, brain-derived neurotrophic factor) remain comparatively underexplored in youth field studies, reflecting methodological constraints rather than theoretical irrelevance (25).

### 4.4 Inequity and the macrosystem

Fourth, cross-sectional and meta-analytic evidence converge on the conclusion that macro- and chronosystem influences (national policy, sociocultural norms, weather, geography) produce associations with youth PA that are at least as large as microsystem effects (17). Yet supportive built environments remain inequitably distributed, both within and between countries. The WHO GAPPA framework (2) offers a coherent policy architecture, but translation into sustained school-based programs, culturally appropriate family interventions, and equitable urban design remains uneven, especially in low- and middle-income settings (3).

### 4.5 Evidence balance, recent literature, and causal interpretation

The present synthesis should be interpreted in light of marked evidence imbalance across SEM levels. Intrapersonal and interpersonal correlates were supported by the largest number of records, including nationally representative surveys, longitudinal data, and several meta-analyses. Organizational evidence was more heterogeneous because school-based and after-school programs differed in duration, dose, implementation fidelity, and psychosocial content. Community-level evidence was relatively consistent for built-environment correlates of PA, but less direct for flourishing and depression. Policy-level evidence was the sparsest and most distal; therefore, policy findings are best understood as contextual associations and plausible intervention targets rather than evidence of direct causation.

Recent publications from 2025 and 2026 reinforce and refine these conclusions. Zhao et al. (34) used an SEM-guided meta-analysis to show that children's and adolescents' PA is associated with multilevel correlates, supporting the value of ecological synthesis while underscoring heterogeneity across levels. Fu et al. (35) found that PA interventions were associated with improved mental-health outcomes in typically developing youth but emphasized low certainty due to methodological limitations. Viskari et al. (36) similarly showed that school-based PA promotion has potential mental-health relevance while highlighting the need for more robust long-term and implementation-sensitive evidence. Du et al. (37) reported a small beneficial association of aerobic exercise with adolescent depressive symptoms and identified a nonlinear dose-response pattern, whereas Liu et al. (38) found small but significant overall intervention effects and suggested that baseline psychological risk and intervention type may moderate outcomes. Collectively, these recent findings support the multilevel framing of the present review while reinforcing the need for cautious causal language and stronger longitudinal, experimental, and policy-evaluation designs.

### 4.6 Implications for practice and policy

Practical implications coalesce into three priorities. Educational practitioners should prioritize PE curricula that cultivate motor competence, self-efficacy, and positive peer interaction, intrapersonal and interpersonal capacities that are consistently associated with PA and flourishing. Family-level interventions should equip parents with logistic-support skills, including transport to sport, enrolment, and co-participation, rather than relying solely on passive modeling. Community and policy actors should invest in walkable neighborhoods, proximate recreation facilities, and safe active-travel infrastructure, recognizing that these structural investments may support PA participation and positive mental health over the developmental arc.

### 4.7 Limitations

This review has several limitations. The predominance of cross-sectional designs precludes causal inference, and self-report PA measurement may overestimate activity, biasing correlates downward. The evidence base was also imbalanced across SEM strata: intrapersonal and interpersonal correlates were comparatively well represented, whereas organizational, community, and especially policy-level correlates were less frequently examined and less amenable to direct causal interpretation. Included studies varied in flourishing and depression instrumentation, complicating direct comparison. Only English-language sources were searched; although this is a common constraint, it may introduce cultural bias. Formal GRADE certainty assessment and quantitative reporting-bias analysis were not conducted because the review prioritized narrative synthesis over a new meta-analysis. Finally, the five-level SEM, while analytically clarifying, risks obscuring cross-level feedback loops that dynamic-systems frameworks such as Bronfenbrenner's bioecological PPCT model (8, 17) more accurately represent.

A further review-process limitation is that screening, data extraction, and methodological appraisal were conducted by a single reviewer. This may have increased susceptibility to selection, extraction, and appraisal error, particularly for records with ambiguous inclusion boundaries or for tools requiring judgment-based ratings. To mitigate this constraint, the author used prespecified eligibility criteria, repeated internal consistency checks, standardized extraction fields, and transparent reporting of study-level appraisal results in the Supplementary material; nevertheless, the internal validity of the synthesis should be interpreted with this limitation in mind.

### 4.8 Future research directions

Future research should prioritize longitudinal designs with objective PA measurement (accelerometry, GPS-enabled devices), inclusion of youth self-reports of flourishing alongside parent-reports, and policy-embedded trials that leverage natural experiments. Multi-level statistical models (e.g., hierarchical linear models, cross-classified random-effects models) can quantify the relative contribution of each SEM stratum. Finally, given the digital restructuring of adolescence, research should interrogate how social media, e-sports, and gamified PA environments reshape traditional socioecological correlates (3).

## 5 Conclusion

Youth PA, flourishing, and depressive symptomatology are associated with correlates nested across five socioecological levels. Evidence indicates that intrapersonal strengths, family and peer support, school environments, community infrastructure, and national policy are each related to these outcomes, often in ways that may interact across levels. Translating this evidence into practice requires coordinated strategies that simultaneously cultivate psychological resources, strengthen family and peer ecosystems, enhance school PE ecosystems, redesign youth-friendly built environments, and embed equity within national physical-activity and mental-health policies. A coherent socioecological public-health agenda is warranted to help children and adolescents move more, languish less, and flourish in an era of mounting mental-health need, while future causal inference should rely on stronger longitudinal, experimental, and natural-experiment designs.
