# Supplementary material for: Multilevel socioecological correlates of physical activity, flourishing, and depression in children and adolescents: a structured evidence synthesis
Source: Front Public Health. 2026 Sep 3;14:1871867. doi: 10.3389/fpubh.2026.1871867 (PMC13584857; doi:10.3389/fpubh.2026.1871867)
Supplement: Supplementary file 1 [file Data_Sheet_1.pdf]

Supplementary material

Manuscript: Multilevel socioecological correlates of physical activity, flourishing, and depression in children and adolescents: a structured evidence synthesis

Author: TaeEung Kim

Journal: Frontiers in Public Health | Article type: Review

Supplementary Table S1. Characteristics of all included evidence records retained for qualitative synthesis (N = 36)

This table expands the selected-study summary in the main manuscript and is intended for submission as supplementary material. PA, physical activity; MVPA, moderate-to-vigorous physical activity; SEM, socioecological model; AXIS, Appraisal tool for Cross-Sectional Studies; NOS, Newcastle-Ottawa Scale; AMSTAR-2, A MeaSurement Tool to Assess systematic Reviews 2.

**No.**	**Study citation**	**Country / design / sample**	**Age / population**	**SEM level(s) / outcome(s)**	**Principal finding relevant to this review**	**Quality appraisal**
1	Foster, Emick, and Griffith (2024). Physical activity and flourishing in adolescents in a nationally representative sample. Res Pediatr Neonatol.	USA; cross-sectional; National Survey of Children's Health; N = 24,488	Adolescents 12-17 y	Intrapersonal; community / PA and flourishing	Meeting PA guidelines was associated with greater odds of flourishing; inactivity and <=3 active days/week were associated with higher risk of not flourishing.	AXIS: moderate
2	Bates and Hansen (2021). Physical activity participation, flourishing and academic engagement in adolescents with obesity. Pediatr Obes. doi:10.1111/ijpo.12816	USA; cross-sectional; NSCH; N = 4,507	Adolescents 12-17 y with obesity	Intrapersonal / PA, flourishing, academic engagement	Any PA participation, including below guideline levels, was positively associated with flourishing and academic engagement.	AXIS: moderate
3	Bethell, Gombojav, and Whitaker (2019). Family resilience and connection promote flourishing among US children, even amid adversity. Health Aff. doi:10.1377/hlthaff.2018.05425	USA; cross-sectional; NSCH; N = 47,873	Children/adolescents 6-17 y	Interpersonal; community / child flourishing	Family resilience and connection showed graded positive associations with child flourishing across adversity and socioeconomic strata.	AXIS: high
4	Rodriguez-Ayllon et al. (2019). Role of physical activity and sedentary behavior in the mental health of preschoolers, children and adolescents. Sports Med. doi:10.1007/s40279-019-01099-5	International; systematic review and meta-analysis; 114 studies	2-18 y	Intrapersonal; interpersonal / mental health	PA showed beneficial associations with mental health, with pooled effects suggesting small but significant improvements in youth mental-health outcomes.	AMSTAR-2: high
5	Recchia et al. (2023). Physical activity interventions to alleviate depressive symptoms in children and adolescents. JAMA Pediatr. doi:10.1001/jamapediatrics.2022.5090	International; systematic review and meta-analysis; 21 RCTs; N = 2,441	<19 y	Intrapersonal; organizational / depressive symptoms	PA interventions significantly reduced depressive symptoms, with stronger effects among adolescents >=13 y and those with elevated baseline symptoms.	AMSTAR-2: high
6	Wang et al. (2022). Systematic review and meta-analysis of the effects of exercise on depression in adolescents. Child Adolesc Psychiatry Ment Health. doi:10.1186/s13034-022-00453-2	International; systematic review and meta-analysis; 19 RCTs	Adolescents 12-18 y	Intrapersonal / depressive symptoms	Exercise reduced adolescent depressive symptoms; moderate-intensity aerobic exercise delivered several times per week showed comparatively stronger effects.	AMSTAR-2: high
7	Lubans et al. (2016). Physical activity for cognitive and mental health in youth: a systematic review of mechanisms. Pediatrics. doi:10.1542/peds.2016-1642	International; systematic review; 25 articles	<18 y	Intrapersonal; organizational / cognitive and mental health	Physical self-perceptions and self-esteem were among the strongest psychosocial mechanisms linking PA to youth mental-health benefits.	AMSTAR-2: moderate-high
8	Hu et al. (2021). Social-ecological correlates of non-organized physical activity in children and adolescents. Int J Environ Res Public Health. doi:10.3390/ijerph18168815	International; systematic review and meta-analysis; 43 studies	5-18 y	Multi-level SEM / non-organized PA	Ecological correlates were positively associated with non-organized PA; macro/chronosystem factors showed relatively large pooled correlations.	AMSTAR-2: moderate
9	Ornelas, Perreira, and Ayala (2007). Parental influences on adolescent physical activity: a longitudinal study. Int J Behav Nutr Phys Act. doi:10.1186/1479-5868-4-3	USA; longitudinal; Add Health; N = 13,246	Grades 7-12	Interpersonal / MVPA	Family cohesion, parent-child communication, and parental engagement predicted adolescent MVPA; self-esteem partially mediated associations.	NOS: high

No.	Study citation	Country / design / sample	Age / population	SEM level(s) / outcome(s)	Principal finding relevant to this review	Quality appraisal
10	Tsuji, Kunii, and Yamazaki (2023). Parental support for physical activity and children's physical activities. BMC Sports Sci Med Rehabil. doi:10.1186/s13102-023-00703-6	Japan; cross-sectional; N = 717	Children approx. 9 y	Interpersonal; community / MVPA and light PA	Parental logistic support, especially sports-club enrolment, was associated with higher MVPA; community resource use supported walking.	AXIS: moderate
11	Ding, Sallis, Kerr, Lee, and Rosenberg (2011). Neighborhood environment and physical activity among youth: a review. Am J Prev Med. doi:10.1016/j.amepre.2011.06.036	International; systematic review; 103 studies	5-18 y	Community / PA	Walkability, traffic safety, residential density, land-use mix, and recreation-facility access were the most consistent environmental correlates.	AMSTAR-2: moderate
12	Christiana, Bouldin, and Battista (2017). Adolescent physical activity: moderation of individual factors by neighborhood environment. Am J Prev Med. doi:10.1016/j.amepre.2017.04.004	USA; cross-sectional; N = 1,286	Adolescents 12-17 y	Intrapersonal x community / MVPA	Supportive neighborhood environments strengthened associations between psychosocial factors and MVPA.	AXIS: moderate
13	Kepper et al. (2020). Neighborhood built environment and physical activity in children and adolescents. JBI Evid Synth. doi:10.11124/JBISRIR-D-19-00350	International; systematic review; 127 studies	5-18 y	Community / active travel, PA, well-being	Pedestrian infrastructure, lower traffic exposure, and shorter distances to destinations supported active travel and youth PA.	AMSTAR-2: moderate-high
14	Carson et al. (2019). Associations between neighborhood recreation environments and adolescent physical activity. J Phys Act Health. doi:10.1123/jpah.2018-0636	USA; cross-sectional; accelerometer study; N = 928	Adolescents approx. 14 y	Community / accelerometer-measured MVPA	Greater availability of recreation facilities near home was associated with substantially higher daily MVPA.	AXIS: moderate
15	van Sluijs et al. (2021). Physical activity behaviours in adolescence: current evidence and opportunities for intervention. Lancet. doi:10.1016/S0140-6736(21)01259-9	Global; position paper/umbrella synthesis	Adolescents 10-19 y	Organizational; community; policy / PA	Supportive schools, social/digital environments, and multi-utility urban design were identified as priority levers for adolescent PA.	Narrative/umbrella: high relevance
16	Ross, Lenow, Kilts, and Cisler (2021). Physical activity, exercise, and fitness in promoting resilience during adolescence. Biol Psychiatry Cogn Neurosci Neuroimaging. doi:10.1016/j.bpsc.2020.08.005	International; narrative/systematic evidence synthesis	Adolescents	Intrapersonal / resilience, mental well-being, brain development	PA and fitness were linked to resilience-related mental-health and neurodevelopmental pathways, although youth field evidence remains limited.	Narrative synthesis: moderate
17	Biddle, Ciaccioni, Thomas, and Vergeer (2019). Physical activity and mental health in children and adolescents: updated review of reviews. Psychol Sport Exerc. doi:10.1016/j.psychsport.2018.08.011	International; review of reviews	Children/adolescents	Intrapersonal / mental health	Evidence supported beneficial associations between PA and several mental-health outcomes, while causal inference and mechanisms required stronger study designs.	AMSTAR-style: moderate-high
18	Cleland et al. (2010). Predictors of time spent outdoors among children: 5-year longitudinal findings. J Epidemiol Community Health. doi:10.1136/jech.2009.087460	Australia; 5-year longitudinal study	Children	Community; interpersonal / outdoor time and PA opportunity	Outdoor time varied according to individual, family, and environmental predictors	NOS: moderate-high
19	Owen, Smith, Lubans, Ng, and Lonsdale (2014). Self-determined motivation and physical activity in children and adolescents. Prev Med. doi:10.1016/j.ypmed.2014.07.033	International; systematic review and meta-analysis	Children/adolescents	Intrapersonal / motivation and PA	Autonomous motivation was positively associated with PA, supporting self-determination theory as an intrapersonal mechanism.	AMSTAR-2: high
20	Smith et al. (2014). Health benefits of muscular fitness for children and adolescents. Sports Med. doi:10.1007/s40279-014-0196-4	International; systematic review and meta-analysis	Children/adolescents	Intrapersonal / muscular fitness and health indicators	Higher muscular fitness was associated with favorable physical and psychological health indicators, reinforcing broad movement-based health promotion.	AMSTAR-2: high
21	Mota et al. (2005). Physical activity and school recess time. J Sports Sci. doi:10.1080/02640410410001730124	Portugal; observational school-based study	Children	Organizational / recess PA and habitual PA	Playground PA during recess differed by sex and was associated with habitual PA, suggesting recess as a school-based promotion context.	AXIS: moderate
22	Janssen and LeBlanc (2010). Systematic review of health benefits of physical activity and fitness in school-aged children and youth. Int J Behav Nutr Phys Act. doi:10.1186/1479-5868-7-40	International; systematic review	School-aged youth 5-17 y	Intrapersonal / PA, fitness, health indicators	Evidence supported health benefits of PA and fitness across multiple outcomes, including indicators relevant to depression and general well-being.	AMSTAR-2: high
23	Eime et al. (2013). Psychological and social benefits of participation in sport for children and adolescents. Int J Behav Nutr Phys Act. doi:10.1186/1479-5868-10-98	International; systematic review	Children/adolescents	Interpersonal; organizational / sport participation and psychosocial health	Sport participation was associated with social and psychological benefits beyond physical-health effects, particularly through social interaction and belonging.	AMSTAR-2: moderate-high

No.	Study citation	Country / design / sample	Age / population	SEM level(s) / outcome(s)	Principal finding relevant to this review	Quality appraisal
24	Biddle and Asare (2011). Physical activity and mental health in children and adolescents: a review of reviews. Br J Sports Med. doi:10.1136/bjsports-2011-090185	International; review of reviews	Children/adolescents	Intrapersonal / depression, anxiety, self-esteem, cognition	PA showed potential benefits for reduced depression/anxiety and improved self-esteem, although evidence quality varied.	AMSTAR-style: moderate
25	Ahn and Fedewa (2011). Meta-analysis of children's physical activity and mental health. J Pediatr Psychol. doi:10.1093/jpepsy/jsq107	International; meta-analysis; 73 studies	Children/adolescents	Intrapersonal; organizational / mental-health outcomes	PA was associated with favorable mental-health outcomes; effects varied by intervention type, outcome domain, and participant characteristics.	AMSTAR-2: moderate-high
26	Marker, Steele, and Noser (2018). Physical activity and health-related quality of life in children and adolescents. Health Psychol. doi:10.1037/hea0000653	International; systematic review and meta-analysis; 33 studies	Children/adolescents	Intrapersonal / health-related quality of life	Descriptive and intervention evidence indicated small positive associations between PA and youth health-related quality of life.	AMSTAR-2: high
27	McMahon et al. (2017). Physical activity in European adolescents and associations with anxiety, depression and well-being. Eur Child Adolesc Psychiatry. doi:10.1007/s00787-016-0875-9	Europe; cross-sectional multi-country study; SEYLE sample	Adolescents	Intrapersonal; interpersonal / PA, sport, anxiety, depression, well-being	More frequent PA and sport participation were associated with lower anxiety/depression and greater well-being; largest differences occurred between inactive and somewhat active youth.	AXIS: moderate-high
28	Babiss and Gangwisch (2009). Sports participation as a protective factor against depression and suicidal ideation in adolescents. J Dev Behav Pediatr. doi:10.1097/DBP.0b013e3181b33659	USA; cross-sectional/secondary analysis	Adolescents	Interpersonal; organizational / sport, depression, suicidal ideation	Sports participation was associated with lower depression and suicidal ideation, partly through self-esteem and social-support pathways.	AXIS: moderate
29	Kremer et al. (2014). Physical activity, leisure-time screen use and depression among children and young adolescents. J Sci Med Sport. doi:10.1016/j.jsams.2013.03.012	Australia; population-based cross-sectional study	Children and young adolescents	Intrapersonal; family context / PA, screen use, depressive symptoms	Higher PA and lower leisure-time screen use were independently associated with lower odds of depressive symptoms.	AXIS: moderate
30	McPhie and Rawana (2015). The effect of physical activity on depression in adolescence and emerging adulthood: a growth-curve analysis. J Adolesc. doi:10.1016/j.adolescence.2015.01.008	Longitudinal secondary analysis	Adolescents to emerging adults	Intrapersonal / PA and depressive-symptom trajectories	PA was examined as a predictor of depressive-symptom trajectories across adolescence and emerging adulthood, supporting developmental timing considerations.	NOS: moderate
31	Brown et al. (2013). Physical activity interventions and depression in children and adolescents: a systematic review and meta-analysis. Sports Med. doi:10.1007/s40279-012-0015-8	International; systematic review and meta-analysis	Children/adolescents	Intrapersonal; organizational / depressive symptoms	PA interventions showed promise for reducing depressive symptoms, but heterogeneity and small samples limited certainty.	AMSTAR-2: moderate
32	Korczak, Madigan, and Colasanto (2017). Children's physical activity and depression: a meta-analysis. Pediatrics. doi:10.1542/peds.2016-2266	International; systematic review and meta-analysis	Children/adolescents	Intrapersonal / PA and depression	Greater PA was associated with lower depressive symptoms, strengthening evidence for PA as a youth mental-health correlate.	AMSTAR-2: high
33	Larun et al. (2006). Exercise in prevention and treatment of anxiety and depression among children and young people. Cochrane Database Syst Rev. doi:10.1002/14651858.CD004691.pub2	International; Cochrane systematic review	Children/young people	Intrapersonal / anxiety and depression	Exercise showed potential preventive and therapeutic effects for anxiety and depressive symptoms, but evidence was limited by study quality.	Cochrane: moderate
34	Spruit et al. (2016). Effects of physical activity interventions on psychosocial outcomes in adolescents. Clin Psychol Rev. doi:10.1016/j.cpr.2016.03.006	International; meta-analytic review	Adolescents	Intrapersonal; organizational / psychosocial outcomes	PA interventions yielded beneficial psychosocial effects, particularly when intervention context supported motivation and self-regulatory capacities.	AMSTAR-2: moderate-high
35	Martins et al. (2015). Adolescents' perspectives on barriers and facilitators of physical activity. Health Educ Res. doi:10.1093/her/cyv042	International; systematic review of qualitative studies	Adolescents	Interpersonal; organizational; community / PA barriers and facilitators	Perceived competence, family/peer support, school opportunities, safety, and built-environment access emerged as salient barriers and facilitators.	Qualitative synthesis: moderate-high
36	Davison and Lawson (2006). Do attributes in the physical environment influence children's physical activity? Int J Behav Nutr Phys Act. doi:10.1186/1479-5868-3-19	International; systematic review	Children	Community / physical environment and PA	Access to recreation facilities, safe routes, and neighborhood design were associated with child PA, supporting built-environment intervention strategies.	AMSTAR-style: moderate

Supplementary Table S1a. Study-level methodological quality and risk-of-bias assessment for the 36 included evidence records.

No.	Evidence record / design	Primary appraisal tool	Quality classification	Risk-of-bias interpretation
1	Foster, Emick, and Griffith (2024); cross-sectional	AXIS	moderate	Some concerns / moderate
2	Bates and Hansen (2021); cross-sectional	AXIS	moderate	Some concerns / moderate
3	Bethell, Gombojav, and Whitaker (2019); cross-sectional	AXIS	high	Low
4	Rodriguez-Ayllon et al; systematic review and meta-analysis	AMSTAR-2	high	Low
5	Recchia et al; systematic review and meta-analysis	AMSTAR-2	high	Low
6	Wang et al; systematic review and meta-analysis	AMSTAR-2	high	Low
7	Lubans et al; systematic review	AMSTAR-2	moderate-high	Some concerns / moderate
8	Hu et al; systematic review and meta-analysis	AMSTAR-2	moderate	Some concerns / moderate
9	Ornelas, Perreira, and Ayala (2007); longitudinal	NOS	high	Low
10	Tsuji, Kunii, and Yamazaki (2023); cross-sectional	AXIS	moderate	Some concerns / moderate
11	Ding, Sallis, Kerr, Lee, and Rosenberg (2011); systematic review	AMSTAR-2	moderate	Some concerns / moderate
12	Christiana, Bouldin, and Battista (2017); cross-sectional	AXIS	moderate	Some concerns / moderate
13	Kepper et al; systematic review	AMSTAR-2	moderate-high	Some concerns / moderate
14	Carson et al; cross-sectional	AXIS	moderate	Some concerns / moderate
15	van Sluijs et al; position paper/umbrella synthesis	Narrative/umbrella	high relevance	Low
16	Ross, Lenow, Kilts, and Cisler (2021); narrative/systematic evidence synthesis	Narrative synthesis	moderate	Some concerns / moderate
17	Biddle, Ciaccioni, Thomas, and Vergeer (2019); review of reviews	AMSTAR-style	moderate-high	Some concerns / moderate
18	Cleland et al; 5-year longitudinal study	NOS	moderate-high	Some concerns / moderate
19	Owen, Smith, Lubans, Ng, and Lonsdale (2014); systematic review and meta-analysis	AMSTAR-2	high	Low
20	Smith et al; systematic review and meta-analysis	AMSTAR-2	high	Low

No.	Evidence record / design	Primary appraisal tool	Quality classification	Risk-of-bias interpretation
21	Mota et al; observational school-based study	AXIS	moderate	Some concerns / moderate
22	Janssen and LeBlanc (2010); systematic review	AMSTAR-2	high	Low
23	Eime et al; systematic review	AMSTAR-2	moderate-high	Some concerns / moderate
24	Biddle and Asare (2011); review of reviews	AMSTAR-style	moderate	Some concerns / moderate
25	Ahn and Fedewa (2011); meta-analysis	AMSTAR-2	moderate-high	Some concerns / moderate
26	Marker, Steele, and Noser (2018); systematic review and meta-analysis	AMSTAR-2	high	Low
27	McMahon et al; cross-sectional multi-country study	AXIS	moderate-high	Some concerns / moderate
28	Babiss and Gangwisch (2009); cross-sectional/secondary analysis	AXIS	moderate	Some concerns / moderate
29	Kremer et al; population-based cross-sectional study	AXIS	moderate	Some concerns / moderate
30	McPhie and Rawana (2015); Longitudinal secondary analysis	NOS	moderate	Some concerns / moderate
31	Brown et al; systematic review and meta-analysis	AMSTAR-2	moderate	Some concerns / moderate
32	Korczak, Madigan, and Colasanto (2017); systematic review and meta-analysis	AMSTAR-2	high	Low
33	Larun et al; Cochrane systematic review	Cochrane	moderate	Some concerns / moderate
34	Spruit et al; meta-analytic review	AMSTAR-2	moderate-high	Some concerns / moderate
35	Martins et al; systematic review of qualitative studies	Qualitative synthesis	moderate-high	Some concerns / moderate
36	Davison and Lawson (2006); systematic review	AMSTAR-style	moderate	Some concerns / moderate

Supplementary Table S1b. Evidence density and interpretive strength by socioecological model level.

SEM level	Relative evidence density	Dominant evidence types	Interpretive implication
Intrapersonal	High	Large surveys, longitudinal analyses, intervention trials, and meta-analyses	Most robust level for PA and mental-health associations, although causality remains limited by design heterogeneity.

SEM level	Relative evidence density	Dominant evidence types	Interpretive implication
Interpersonal	High to moderate	Family, peer, and support studies; cohort and cross-sectional records	Consistent associations with PA and flourishing; mediation and moderation require stronger longitudinal testing.
Organizational / school	Moderate	School, PE, recess, and after-school sport evidence	Program effects appear context- and implementation-sensitive; dose and fidelity should be interpreted cautiously.
Community / built environment	Moderate to limited for mental health	Built-environment reviews and accelerometer studies	Evidence is clearer for PA than for flourishing or depression; equity and access remain important contextual issues.
Public policy	Limited	Guidelines, surveillance syntheses, and umbrella/position evidence	Policy findings are distal and hypothesis-generating; direct causal inference is not warranted.

13

Supplementary Appendix S1. PRISMA-informed reporting checklist and database-specific search strategy

The checklist is provided as a transparency aid for this structured evidence synthesis and is not intended to imply prospective registration or submission as a registered systematic review. Checklist items are mapped to the Frontiers-formatted manuscript. Page and line locations should be checked against the final submission PDF generated by the Frontiers submission system. The revised supplementary material also reports study-level quality appraisal, SEM evidence-density imbalance, and the recent-literature update.

Supplementary Table S2. PRISMA-informed reporting checklist.

Item	Section / topic	Reporting requirement	Location in manuscript	Status / note
1	Title	Identify the report as a structured evidence synthesis / Review and avoid implying prospective registration.	Title page, p. 1	Completed; title revised to structured evidence synthesis.
2	Abstract	Provide a structured abstract summarizing background, objectives, eligibility criteria, sources, appraisal, synthesis, results, limitations, and conclusions.	Abstract, p. 2	Completed; abstract clarifies that the evidence synthesis used PRISMA-informed reporting items and was not prospectively registered.
3	Rationale	Describe the rationale for the review in the context of existing knowledge.	Introduction, pp. 3-4	Completed
4	Objectives	Provide an explicit statement of review objectives/questions.	Introduction, p. 4	Completed
5	Eligibility criteria	Specify inclusion and exclusion criteria and how studies were grouped for synthesis.	Methods 2.2, pp. 4-5; Appendix B	Completed
6	Information sources	Specify all databases, registers, websites, organizations, reference lists, and dates searched.	Methods 2.3, pp. 4-5; Appendix B	Completed; exact export dates should be retained in author files.
7	Search strategy	Present full search strategies for all databases and filters/limits used.	Appendix B	Completed
8	Selection process	Describe how records and reports were screened, including the number of reviewers and automation tools.	Methods 2.4, p. 5	Completed; single-author screening was transparently reported, and mitigation procedures are described in the Methods and Limitations.
9	Data collection process	Describe how data were extracted and whether forms were piloted/checked.	Methods 2.4, p. 5	Completed; standardized extraction fields were used and rechecked by the author.
10	Data items	List and define all outcomes and variables sought, including assumptions and simplifications.	Methods 2.2-2.4, pp. 4-5; Supplementary Table S1	Completed
11	Risk of bias in individual studies	Specify tools and procedures used to assess risk of bias or methodological quality.	Methods 2.5; Results 3.2; Table 4; Supplementary Table S1a	Completed; design-appropriate tools and study-level classifications are reported.
12	Effect measures	Specify effect measures used for each outcome or synthesis.	Methods 2.6, p. 5; Results 3.2-3.6	Partly completed; narrative review reports effect sizes descriptively from prior meta-analyses.
13	Synthesis methods	Describe eligibility for synthesis, preparation of data, tabulation, and synthesis model/method.	Methods 2.6, p. 5	Completed
14	Reporting bias assessment	Describe methods used to assess risk of bias due to missing results.	Limitations, pp. 16-17	Partly completed; quantitative publication-bias assessment was not undertaken because a new meta-analysis was not performed.
15	Certainty assessment	Describe methods used to assess certainty/confidence in the body of evidence.	Table 4; Supplementary Table S1a; Discussion 4.7	Partly completed; formal GRADE was not conducted because the review used narrative synthesis across heterogeneous evidence records.
16	Study selection results	Report numbers screened, assessed, excluded, and included, with reasons.	Figure 1, p. 6; Results 3.1, p. 9	Completed
17	Study characteristics	Cite each included study and present its characteristics.	Table 2; Supplementary Table S1	Completed with full supplementary table.
18	Risk of bias results	Present risk-of-bias or methodological-quality assessment for included studies.	Results 3.2; Table 4; Supplementary Table S1 and S1a	Completed at design-level and study-level summary.
19	Results of individual studies	Present results for all outcomes for each study or study group.	Results 3.2-3.6; Supplementary Table S1	Completed as narrative synthesis.

Item	Section / topic	Reporting requirement	Location in manuscript	Status / note
20	Synthesis results	Present results of each synthesis and investigations of heterogeneity.	Results 3.2-3.6; Table 3	Completed narratively; heterogeneity handled descriptively.
21	Reporting bias results	Present assessments of risk of bias due to missing results.	Discussion 4.6	Not formally assessed; limitation stated because the synthesis was narrative and did not compute a new pooled effect.
22	Certainty of evidence	Present certainty or confidence in the body of evidence.	Table 4; Supplementary Table S1a; Discussion 4.7	Partly completed; confidence in evidence is discussed by study design and SEM level.
23	Discussion	Provide general interpretation, limitations of evidence, review-process limitations, and implications.	Discussion, pp. 15-17	Completed
24	Registration and protocol	Provide registration information or state that the review was not registered.	Methods 2.1; Supplementary Table S2	Completed; the review was not prospectively registered in PROSPERO, OSF, INPLASY, or another registry. The manuscript is framed as a structured evidence synthesis rather than a registered systematic review.
25	Support	Describe funding or other support and role of funders.	Declarations, p. 17	Completed
26	Competing interests	Declare competing interests of review authors.	Declarations, p. 17	Completed
27	Availability of data, code, and materials	Report availability of templates, data extracted, analytic code, and other materials.	Data availability, p. 18; Supplementary Table S1; Appendix B	Completed for extracted public data and search materials.

Supplementary Table S3. PubMed search strategy.

**Search string**	**((child*[Title/Abstract] OR adolescent*[Title/Abstract] OR youth[Title/Abstract] OR teen*[Title/Abstract] OR "school-aged"[Title/Abstract]) AND ("physical activity"[Title/Abstract] OR exercise[Title/Abstract] OR sport*[Title/Abstract] OR MVPA[Title/Abstract]) AND (flourish*[Title/Abstract] OR "well-being"[Title/Abstract] OR wellbeing[Title/Abstract] OR "mental health"[Title/Abstract] OR depress*[Title/Abstract] OR "depressive symptoms"[Title/Abstract]) AND (socioecological[Title/Abstract] OR "social-ecological"[Title/Abstract] OR Bronfenbrenner[Title/Abstract] OR "social support"[Title/Abstract] OR parent*[Title/Abstract] OR peer*[Title/Abstract] OR school[Title/Abstract] OR "physical education"[Title/Abstract] OR neighborhood[Title/Abstract] OR "built environment"[Title/Abstract] OR policy[Title/Abstract]))**
Limits / filters	Humans; English; publication dates 1 Jan 2000 to 30 Apr 2026; age approximately 6-18 years.

Supplementary Table S4. Scopus search strategy.

**Search string**	**TITLE-ABS-KEY(child* OR adolescent* OR youth OR teen* OR "school-aged") AND TITLE-ABS-KEY("physical activity" OR exercise OR sport* OR MVPA) AND TITLE-ABS-KEY(flourish* OR "well-being" OR wellbeing OR "mental health" OR depress* OR "depressive symptoms") AND TITLE-ABS-KEY(socioecological OR "social-ecological" OR Bronfenbrenner OR "social support" OR parent* OR peer* OR school OR "physical education" OR neighborhood OR "built environment" OR policy)**
Limits / filters	Peer-reviewed journal articles; English; 2000-April 2026; human studies; child/adolescent populations.

Supplementary Table S5. Web of Science Core Collection search strategy.

**Search string**	**TS=(child* OR adolescent* OR youth OR teen* OR "school-aged") AND TS=("physical activity" OR exercise OR sport* OR MVPA) AND TS=(flourish* OR "well-being" OR wellbeing OR "mental health" OR depress* OR "depressive symptoms") AND TS=(socioecological OR "social-ecological" OR Bronfenbrenner OR "social support" OR parent* OR peer* OR school OR "physical education" OR neighborhood OR "built environment" OR policy)**
Limits / filters	Document types: article or review; language: English; publication years: 2000-April 2026.

Supplementary Table S6. PsycINFO search strategy.

**Search string**	**(child* OR adolescent* OR youth OR teen* OR "school-aged") AND ("physical activity" OR exercise OR sport* OR MVPA) AND (flourish* OR "well-being" OR wellbeing OR "mental health" OR depress* OR "depressive symptoms") AND (socioecological OR "social-ecological" OR Bronfenbrenner OR "social support" OR parent* OR peer* OR school OR "physical education" OR neighborhood OR "built environment" OR policy)**
Limits / filters	Peer-reviewed journal articles; English; human; childhood/adolescence age filters where available; 2000-April 2026.

Supplementary Table S7. SPORTDiscus search strategy.

**Search string**	**(child* OR adolescent* OR youth OR teen* OR "school-aged") AND ("physical activity" OR exercise OR sport* OR MVPA) AND (flourish* OR "well-being" OR wellbeing OR "mental health" OR depress* OR "depressive symptoms") AND (socioecological OR "social-ecological" OR Bronfenbrenner OR "social support" OR parent* OR peer* OR school OR "physical education" OR neighborhood OR "built environment" OR policy)**
Limits / filters	Scholarly peer-reviewed journals; English; 2000-April 2026; youth samples.

Supplementary Table S8. Eligibility and extraction framework.

**Domain**	**Operational definition**
Population	Children and adolescents aged approximately 6-18 years; school-aged youth samples were retained when youth data were extractable.
Exposure/correlate	At least one SEM-level correlate: intrapersonal, interpersonal, organizational/school, community/built environment, or public policy.
Outcomes	Physical activity, exercise, MVPA, sport participation, flourishing, well-being, depression, depressive symptoms, or closely related youth mental-health indicators.
Designs	Observational, longitudinal, intervention, quasi-experimental, systematic review, meta-analysis, umbrella/position synthesis, or mixed-method evidence records relevant to the review question.
Exclusions	Editorials, dissertations, conference abstracts, non-English full texts, non-youth samples, clinical-only samples without extractable youth data, and reports without SEM-level correlates.
Extracted fields	Bibliographic identifiers, country, design, sample size, age range, SEM level, exposure/correlate, outcome, main finding, and quality-appraisal summary.

Supplementary Table S9. Recent 2025-2026 literature incorporated into the revised Discussion.

**Citation**	**Evidence type**	**Relevance to revision**
Zhao et al. (2025)	SEM-guided systematic review and meta-analysis	Supports multilevel PA correlates in youth and reinforces evidence imbalance across ecological levels.
Fu et al. (2025)	Systematic review and meta-analysis	Updates intervention evidence for typically developing children and adolescents and emphasizes low certainty.
Viskari et al. (2025)	School-based PA systematic review	Contextualizes organizational/school-level evidence and implementation limitations.
Du et al. (2026)	Bayesian dose-response meta-analysis	Updates adolescent depression evidence and cautions against linear dose assumptions.
Liu et al. (2026)	Systematic review and meta-analysis	Updates intervention-type and baseline-risk moderators for youth mental health outcomes.

Supplementary appendix references

Page MJ, McKenzie JE, Bossuyt PM, et al. The PRISMA 2020 statement: an updated guideline for reporting systematic reviews. BMJ (2021) 372:n71. doi: 10.1136/bmj.n71

Page MJ, Moher D, Bossuyt PM, et al. PRISMA 2020 explanation and elaboration: updated guidance and exemplars for reporting systematic reviews. BMJ (2021) 372:n160. doi: 10.1136/bmj.n160

Zhao C, Yuan J, Huang WW. Multilevel determinants of physical activity in children and adolescents: a meta-analysis guided by social ecological model. BMC Sports Sci Med Rehabil. 2025;17:202. doi:10.1186/s13102-025-01225-z

Fu Q, Li L, Li Q, Wang J. The effects of physical activity on the mental health of typically developing children and adolescents: a systematic review and meta-analysis. BMC Public Health. 2025;25:1514. doi:10.1186/s12889-025-22690-8

Viskari T, Koivumäki T, Appelqvist-Schmidlechner K, Ståhl T, Ruiz-Ariza A, Fröjd S. The effectiveness of school-based physical activity promotion on mental health among children and adolescents: a systematic review. Scand J Med Sci Sports. 2025;35(10):e70150. doi:10.1111/sms.70150

Du H, Dong D, Huang H. Aerobic exercise for depression symptoms in adolescents: a systematic review and dose-response meta-analysis. BMC Public Health. 2026;26:828. doi:10.1186/s12889-026-26504-3

Liu Q, Xu Z, Tai P, Lian Z, Cheng W, Zhang J. Effects of physical activity interventions on mental health in children and adolescents: a systematic review and meta-analysis of intervention type and baseline risk as moderators. Front Psychol. 2026;17:1825603. doi:10.3389/fpsyg.2026.1825603
